# Cold temperature enhances innate eosinophilic airway inflammation via transient receptor potential ankyrin1

**DOI:** 10.3389/fimmu.2025.1655919

**Published:** 2025-11-13

**Authors:** Yoichi Dotake, Takahiro Matsuyama, Kentaro Machida, Hiromi Matsuyama, Koichi Takagi, Keiko Mizuno, Keiko Kan-o, Tomoyuki Kuwaki, Hiromasa Inoue, Kentaro Tanaka

**Affiliations:** 1 Department of Pulmonary Medicine, Graduate School of Medical and Dental Sciences, Kagoshima University, Kagoshima, Japan; 2 Department of Respiratory Medicine, Graduate School of Medical Sciences, Kyushu University, Fukuoka, Japan; 3 Department of Respiratory Medicine, Tokyo Women’s Medical University, Tokyo, Japan; 4 Department of Physiology, Graduate School of Medical and Dental Sciences, Kagoshima University, Kagoshima, Japan

**Keywords:** asthma, airway inflammation, group 2 innate lymphoid cell, transient receptor potential A1, thymic stromal lymphopoietin

## Abstract

**Background and objective:**

Asthma exacerbations due to cold air exposure are well recognized; however, the underlying mechanisms remain unclear. We investigate the role of the transient receptor potential ankyrin1 (TRPA1) channel in cold air-induced aggravation of innate airway inflammation using a murine model of papain stimulation combined with cold air exposure.

**Methods:**

Wild-type (WT) and *Trpa1* knockout (KO) mice were treated intranasally with papain under different temperature conditions. Bronchoalveolar lavage fluid (BALF) and lung tissues were analyzed. The effects of the TRPA1 antagonist HC030031 were also evaluated. Additionally, human bronchial epithelial (HBE) cells were stimulated with papain and the TRPA1 agonist allyl isothiocyanate (AITC).

**Results:**

Papain treatment increased eosinophils in BALF, and the number of eosinophils was similar in WT and *Trpa1* KO mice. Papain treatment with cold air exposure in WT mice significantly increased the number of eosinophils and type-2 innate lymphoid cells and the protein expressions of IL-5, IL-13, and TSLP in BALF. However, cold air exposure failed to augment airway eosinophilia in response to papain in *Trpa1* KO mice. Treatment of HC030031 replicated the findings observed in *Trpa1* KO mice. AITC enhanced papain-induced TSLP production in HBE cells by increasing the intracellular calcium concentration.

**Conclusions:**

These findings suggest that TRPA1 channels expressed in airway epithelial cells play a critical role in producing TSLP, contributing to the enhancement of eosinophilic airway inflammation mediated by innate immunity upon cold air exposure, providing valuable insights into the mechanisms underlying asthma exacerbation triggered by cold temperatures.

## Introduction

Asthma is a heterogeneous disease, characterized by chronic airway inflammation and variable airflow obstruction, that causes dyspnea, cough, and wheezing (1–3). Asthma therapy has advanced remarkably, and many asthmatic patients are able to achieve good disease control with current therapies. However, a substantial number of patients experience asthma exacerbations by viral infections and environmental risk factors, which can be life-threatening and a significant burden for them. These can be either antigen-specific stimuli, such as allergens, or antigen-nonspecific stimuli, such as cold air and pollutants, which cause airway inflammation through distinct immune mechanisms (3–5). Although previous studies have found an increased risk of asthma exacerbations or hospital admissions during cold spells (6–8), pathophysiological mechanisms underlying cold air-induced airway responses in asthma remain incompletely understood.

Recent advances in asthma have led to the identification of two major endotypes based on the level of type-2 cytokine-driven inflammation: type-2 high and type-2 low (9). In patients with type-2 high asthma, the type-2 immune response is excessively activated, and T helper type-2 (Th2) cells and group 2 innate lymphoid cells (ILC2s) produce large amounts of the type-2 cytokines like interleukin-4 (IL-4), IL-5, IL-9, and IL-13, which in turn induce asthmatic features (9, 10). Asthma exacerbations are associated with exposure to the external stimuli described above, which trigger the release of epithelial-derived inflammatory ‘alarmin’ cytokines, such as thymic stromal lymphopoietin (TSLP), IL-25, and IL-33. Then, by stimulation with these epithelial-derived cytokines, ILC2s release type-2 cytokines, IL-5, and IL-13. Therefore, ILC2s are considered to be involved in the pathophysiology of asthma exacerbation caused by antigen non-specific stimuli (11, 12).

Transient receptor potential (TRP) channels are multifunctional signaling molecules with many roles in sensory perception and cellular physiology (13). The TRPA1 channel, a member of the TRP subfamily, is widely expressed in sensory nerve endings and in non-neuronal cells. TRPA1 channels have been reported to be activated by cold, heat, and mechanical stimuli (14). The activation of TRPA1 channels leads to coughing in humans (15). A previous study using ovalbumin (OVA)-induced asthma models demonstrated that TRPA1 activation leads to the release of neuropeptides such as calcitonin gene-related peptide (CGRP), substance P, and neurokinin A, which, in turn, promotes the activation of adaptive immunity and cytokine production (16). However, the role of TRPA1 channels in innate immunity-mediated airway inflammation remains unknown.

TRPA1 channels are activated by temperatures below 17°C (17). Therefore, we hypothesized that cold air exposure could enhance ILC2-dependent airway inflammation through TRPA1 channels. In white adipose tissues, cold exposure has been shown to induce the expression of IL-33 and the population of ILC2s and eosinophils (18). Given that cold air-induced exacerbation of asthma is considered to contribute to the innate immune response, we used papain, a plant-derived cysteine protease, as the stimulus in our study. Intranasal papain administration is known to be a typical model of type-2 airway inflammation mediated by innate immunity (19). Papain releases IL-33, IL-25, and TSLP from airway epithelial cells, a process that subsequently activates ILC2s to induce eosinophilic airway inflammation through type-2 cytokine production. Here, we showed that eosinophil accumulation and type-2 cytokine production induced by papain are augmented after cold air exposure. We investigated the mechanisms by which cold air exposure modulates ILC2-dependent airway inflammation using *Trpa1* knockout (KO) mice and *in vitro* experiments.

## Materials and methods

### Mice

C57BL/6 mice (8–12 weeks old) were housed under specific pathogen-free (SPF) conditions (12 h light-dark cycle and 22°C). Mice were maintained in ventilated cabinets equipped with HEPA-filtered air supply. Each cage housed up to three mice with wood shavings bedding, and plastic shelters were provided as enrichment. Mice had free access to standard chow and water. *Trpa1* KO mice, obtained from The Jackson Laboratory, were backcrossed with C57BL/6 mice for more than 10 generations as previously described (20), and then used in the experiments. This animal study was approved by the Animal Care and Use Committees of Kagoshima University - approval: MD22084, MD23011. All animal experiments and handling procedures were performed in accordance with institutional guidelines.

### Papain-induced airway inflammation and cold air exposure

Mice were anesthetized with isoflurane and then administered 25 μg papain (Calbiochem, San Diego, CA, USA) in 40 μl saline intranasally for 3 consecutive days. Mice were sacrificed 24 h after the final papain administration to assess airway inflammation as previously described (21).

**Figure 1:**
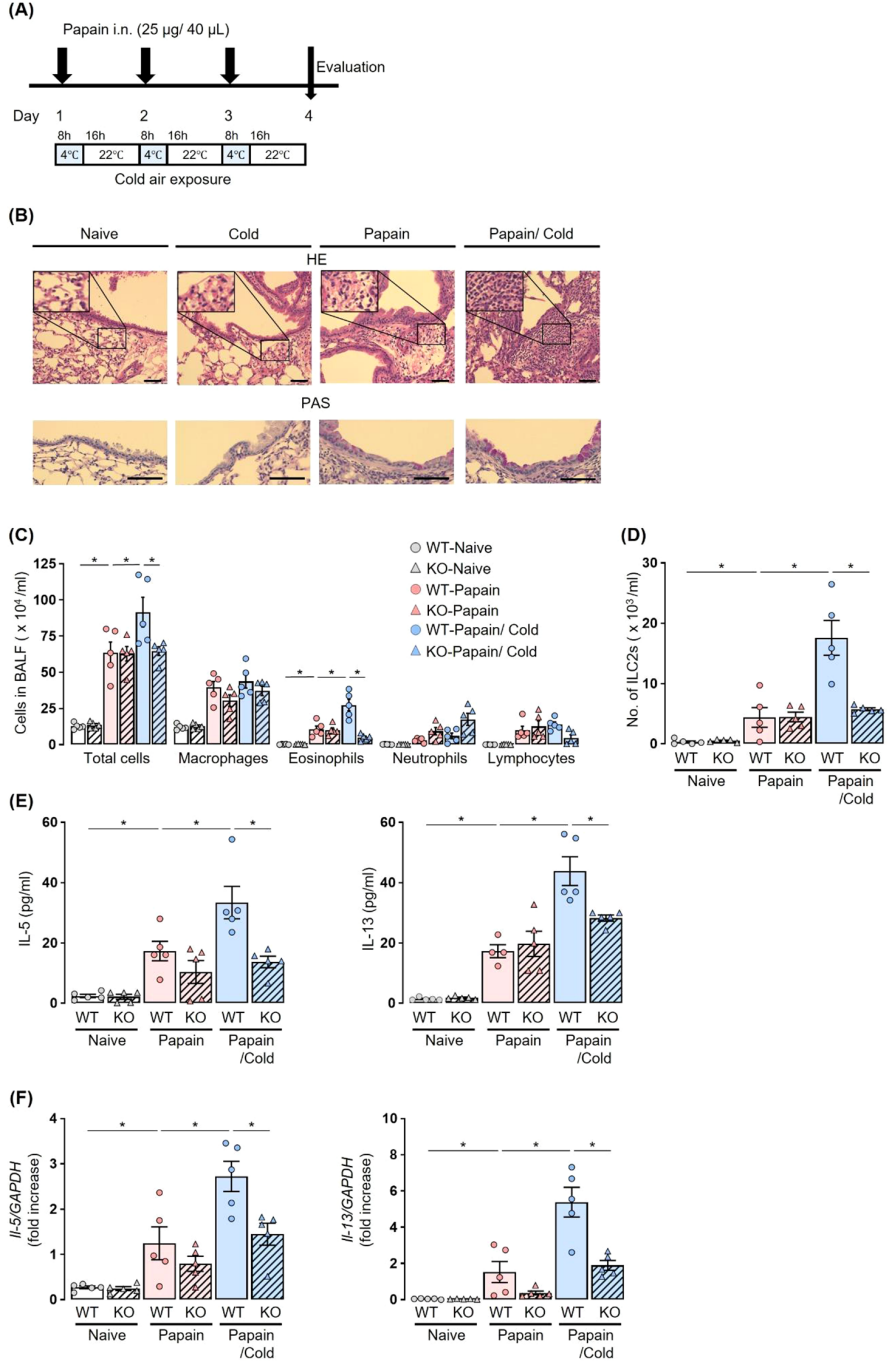
Cold air exposure failed to induce the exacerbation of eosinophilic inflammation in the airway of a papain model of *Trpa1* KO mice. **(A)** Experiment protocol: Airway inflammation was induced by the administration of papain for 3 consecutive days. For cold air exposure, mice were kept at 4°C for 8 h following each papain administration, and then maintained at 22°C for the subsequent 16 h **(B–F)**, Cold air exposure increased airway histological alterations, including epithelial damage, inflammatory cell infiltration, and mucus hypersecretion **(B)**, the numbers of total cells, eosinophils **(C)**, and ILC2s **(D)**, and the production and expression of IL-5 and IL-13 in BALF **(E)** and lungs **(F)** in a papain-treated murine model. Increases in these parameters were suppressed in *Trpa1* KO mice, as compared to WT mice, under cold air exposure. The histology of the airway was examined by hematoxylin-eosin (H&E) (×200 or ×400), and Periodic acid-Schiff (PAS) staining (×400). Scale bar: 100 μm **(B)**. The numbers of ILC2s in BALF were measured using flow cytometry **(D)**. IL-5 and IL-13 production and expression were measured by ELISA **(E)** and real-time PCR **(F)**. Mean ± SEM: n = 4–6 for each group; two-way ANOVA with Tukey’s *post hoc* test; *P < 0.05. Data are representatives of three independent experiments. i.n., intranasal.

For the cold air-exposure experiment, mice were immediately transferred to a 4°C environment for 8 h following each papain administration, and then maintained at 22°C for the subsequent 16 h. Papain administration and cold air exposure were repeated for 3 consecutive days (**Figure 1**).

**Figure 2:**
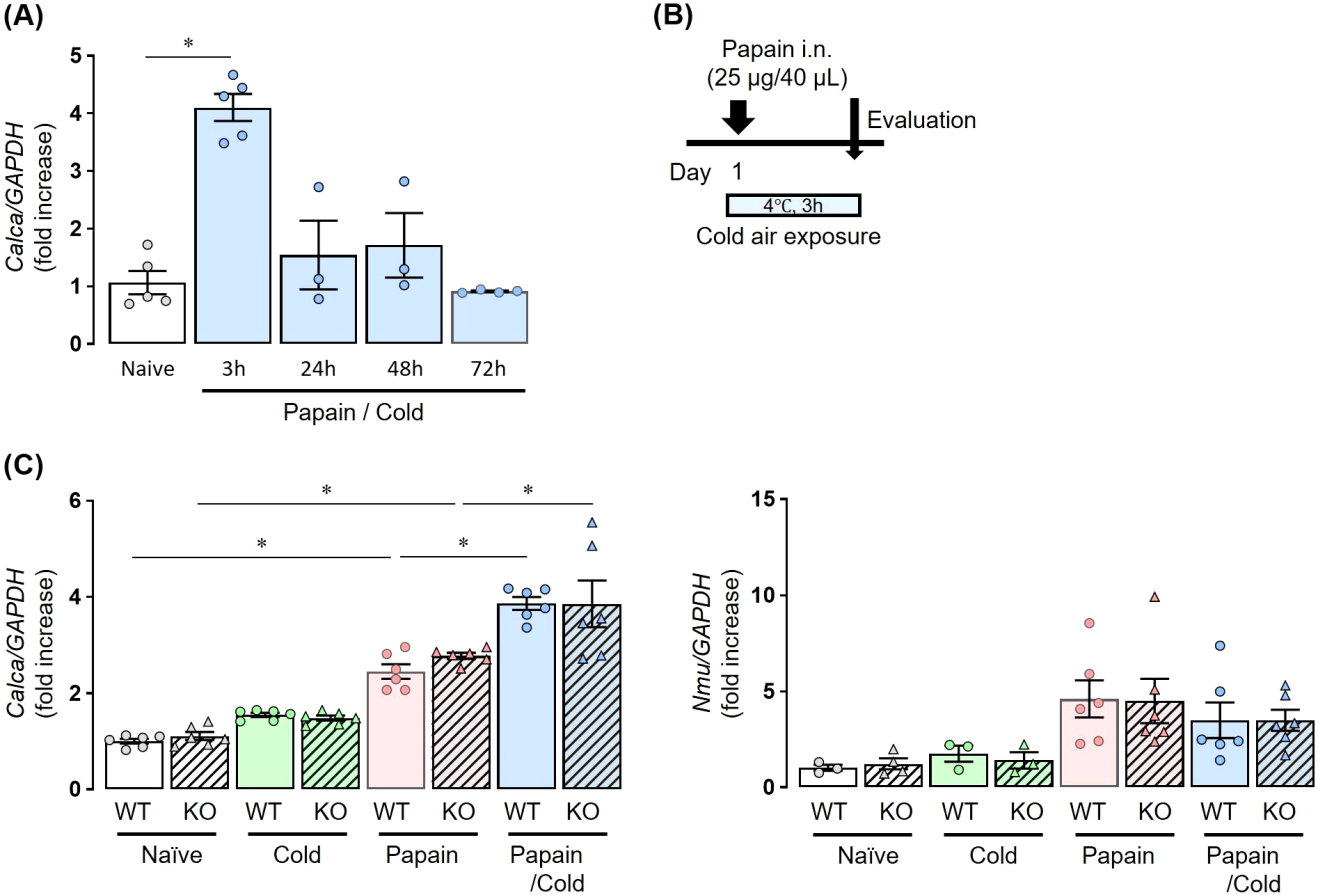
Co-stimulation with papain and cold air exposure enhances neuropeptide expression without Inducing TRPA1 channel activation. **(A)** The expression of *Calca* in papain-treated WT mice exposed to cold air was significantly upregulated compared with naïve mice, peaking at 3 h after treatment. At 24, 48, and 72 h, *Calca* expression declined and was not significantly different from naïve controls. **(B)** Experiment protocol: Mice received a single dose of papain followed by cold exposure for 3 h. **(C)** The expression of *Calca* and *Nmu* mRNA at 3 h after stimulation. *Calca* was upregulated by papain stimulation and further enhanced by the combination with cold air, whereas cold air alone had no effect. This induction occurred irrespective of TRPA1 expression (left). *Calca* and *Nmu* expressions were measured by real-time PCR **(A, B)**. Mean ± SEM: n = 3–5 for each group; two-way ANOVA with Tukey’s *post hoc* test; *P < 0.05. Data are representatives of two independent experiments.

**Figure 3:**
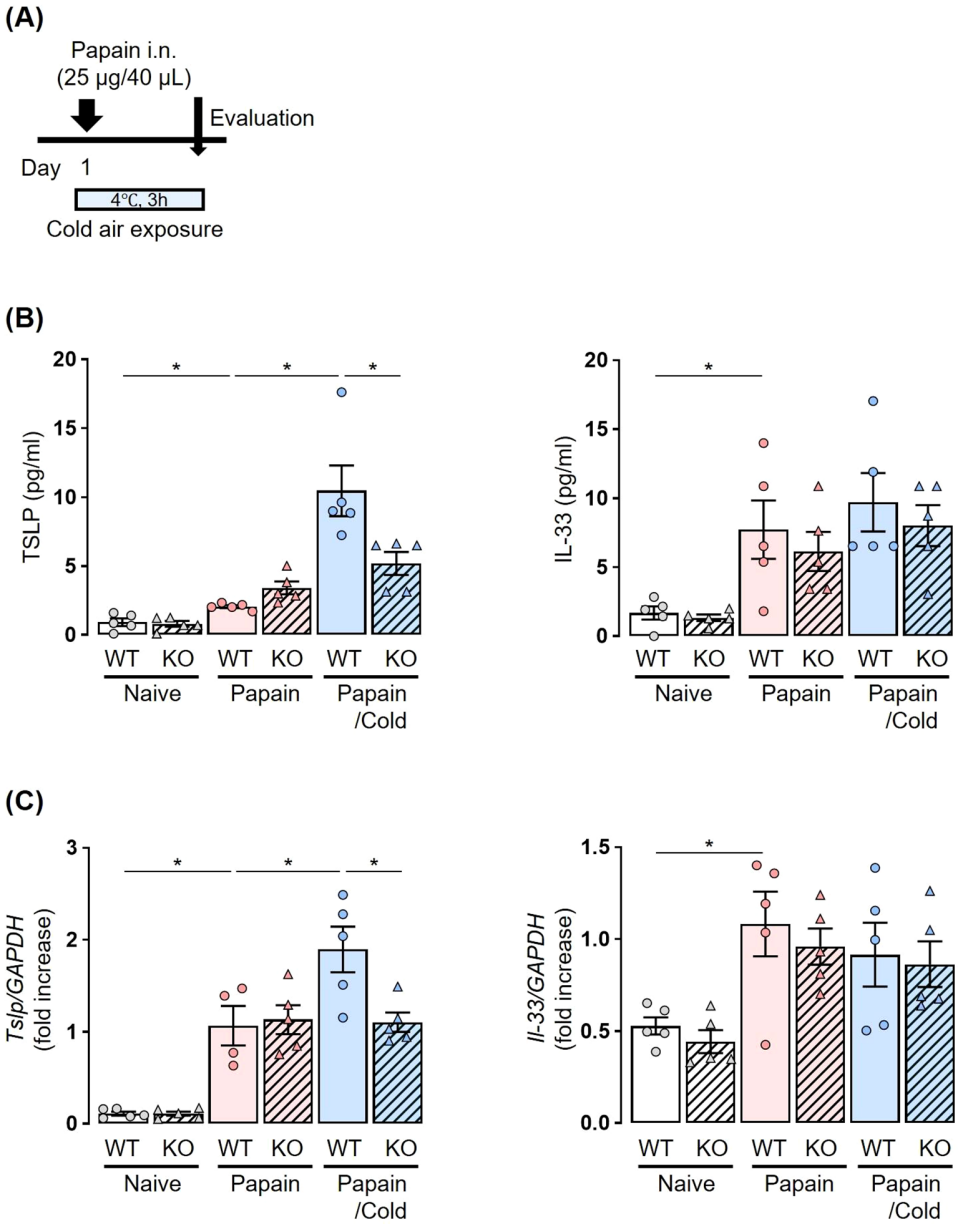
Papain-treated elevation of TSLP levels under cold air exposure is suppressed in *Trpa1* KO mice. **(A)** Experiment protocol: Mice received a single dose of papain followed by cold exposure for 3 h. **(B, C)** Cold air exposure increased the production and expression of TSLP in BALF **(B)** and lungs **(C)** in a papain-treated murine model. No increase in these parameters was observed following cold air exposure in *Trpa1* KO mice. TSLP and IL-33 production and expression were measured by ELISA **(B)** and real-time PCR **(C)**. Mean ± SEM: n = 4–6 for each group; two-way ANOVA with Tukey’s *post hoc* test; *P < 0.05. Data are representatives of three independent experiments.

To analyze neuropeptides and epithelial-derived cytokines, mice were sacrificed after a single papain administration and subsequent exposure to cold air stimuli at 4°C for 3 h (**Figures 2**, **3**).

**Figure 4:**
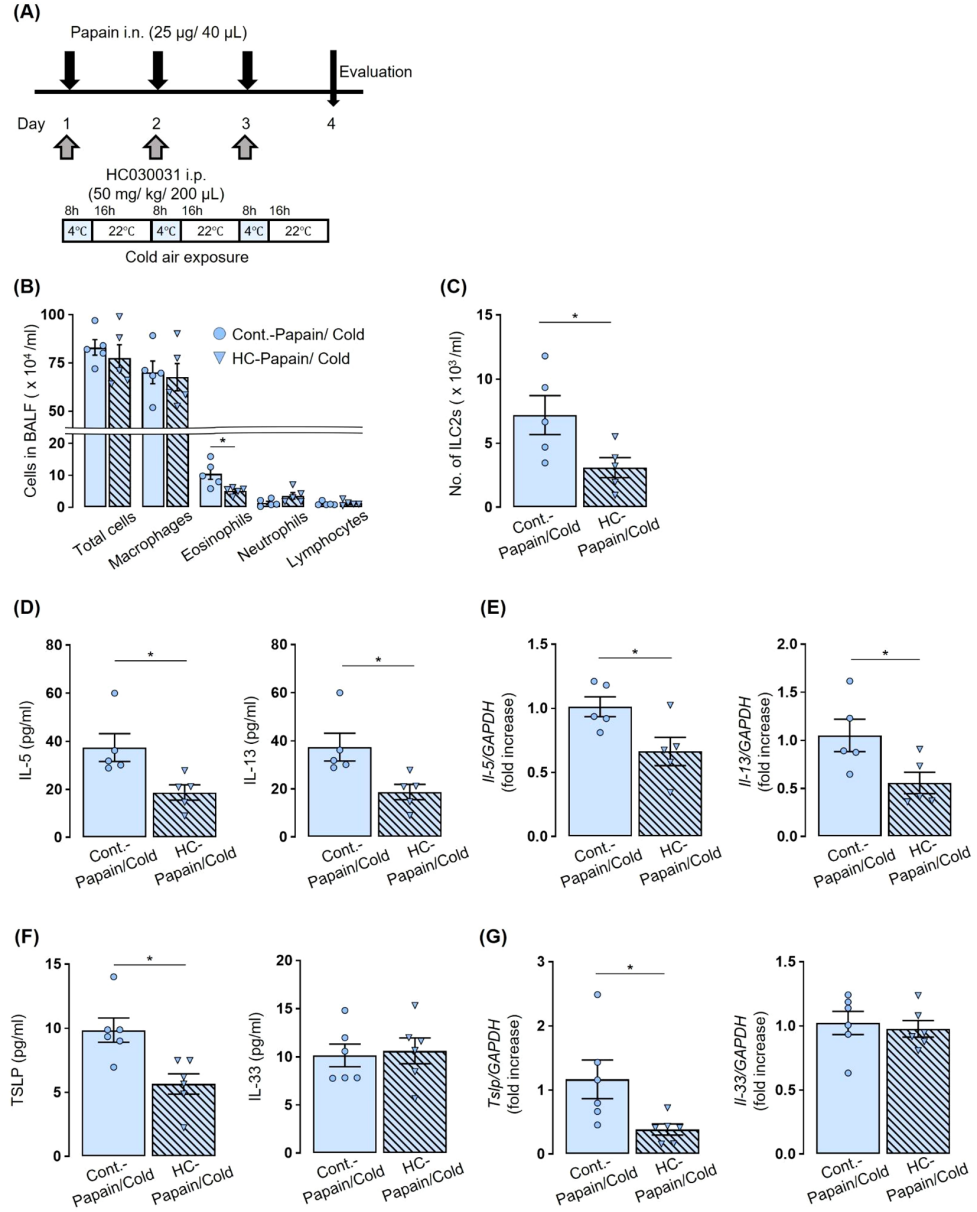
TRPA1 channel antagonist prevented an increase in the number of eosinophils and ILC2s and the production and expression of IL-5, IL-13, and TSLP caused by co-stimulated with papain and cold air exposure. **(A)** Experiment protocol: Mice received intraperitoneal administration of HC-030031–60 min before each papain administration, followed by exposure to cold air. **(B, C)** The administration of HC-030031 prevented the enhanced number of total cells, eosinophils **(B)**, and ILC2s **(C)** in BALF induced by the addition of cold air exposure to papain stimulation. **(D–G)** In mice co-stimulated with papain and cold air exposure, the production and expression of IL-5, IL-13, and TSLP in BALF **(D, F)** and lungs **(E, G)** were prevented by treatment with HC-030031. The number of ILC2s in BALF were measured using flow cytometry **(C)**. IL-5 and IL-13, TSLP, and IL-33 production and expression were measured by ELISA **(D, F)** and real-time PCR **(E, G)**. Mean ± SEM: n = 4–6 for each group; unpaired *t*-tests with Tukey’s *post hoc* test; *P < 0.05. Data are representatives of two independent experiments. HC, HC-030031. i.p., intraperitoneal.

To investigate the pharmacological inhibition of TRPA1 channels, a TRPA1 channel antagonist, HC030031 (Alomone Labs, Jerusalem, Israel), at a dose of 50 mg/kg in 200 μl of PBS, was injected intraperitoneally 60 minutes prior to each papain administration (16) (**Figure 4**).

Bronchoalveolar lavage fluids (BALF) and lung tissues were collected. Total cell counts were determined using a standard hemocytometer. Cytospins (Cytospin 3; Shandon, Pittsburgh, PA, USA) were prepared on glass slides and stained with Diff-Quik (21, 22). Differential cell counts were determined by analyzing 200 cells by conventional morphological criteria (23).

### Primary human bronchial epithelial cell culture and stimulation

Normal human bronchial/tracheal epithelial (NHBE) cells were purchased from Lonza (Walkersville, MD, USA). NHBE cells were incubated with bronchial epithelial growth medium (BEGM; Lonza) with 5% CO_2_ at 37°C. All experiments were conducted using second- or third-passage cells at 70-80% confluency. NHBE cells were seeded at a density of 1 × 10^5^ cells per well in 12-well culture plates (AGC, Tokyo, Japan). The cells were treated individually or in combination for 3–6 h with papain (0.1-10 µM; Sigma-Aldrich, St. Louis, MO, USA), allyl isothiocyanate (10-30 µM; AITC; Sigma-Aldrich), a TRPA1 channel agonist, capsaicin (10-30 µM; Selleck Chemicals, Houston, TX, USA), a TRPV1 channel agonist, and BAPTA-AM (5 µM; Selleck Chemicals), a calcium chelator.

### Measurement of cytokine protein expression by enzyme-linked immunosorbent assays

Each cytokine protein expression in BALF was measured using the Quantikine ELISA Kit (R&D Systems, Minneapolis, MN, USA) in accordance with the manufacturer’s protocol.

### Immunofluorescence staining and enumeration of ILC2s in BALF by flow cytometry

Centrifuged and resuspended cells from BALF were stained for ILC2s with antibodies to surface markers anti-mouse T1/ST2-BV421, anti-mouse KLRG1-BV650, anti-mouse Thy1.2-BV786, anti-mouse CD45.2-PE-Cy7, anti-mouse Lineage Antibody Cocktail (CD3ϵ, CD11b, CD45RA/B220, Ly-76, Ly-6G/Ly-6C)-FITC, or relevant isotype controls, and propidium iodide (PI) was added to remove dead cells (BD Biosciences, San Jose, CA, USA; BioLegend, San Diego, CA, USA). Antibodies used in this study are listed in **Supplementary Table S1** in the Supporting Information.

ILC2s were identified as lineage-negative (Lin^−^) Thy1.2^+^ T1/ST2^+^ KLRG1^+^ CD45.2^+^ cells. ILC2s were enumerated using BD FACSCelesta (BD Biosciences). All data were analyzed using FlowJo software (BD Biosciences).

### Quantitative real-time polymerase chain reaction

Total RNA was extracted from lungs and NHBE cells by using TRI reagent (Molecular Research Center, Inc., Cincinnati, OH, USA), and cDNA was synthesized with the High-Capacity cDNA Reverse Transcription Kit (Life Technologies, Carlsbad, CA, USA). Quantitative PCR was performed with TaqMan PCR using TaqMan Universal PCR Master Mix and the StepOnePlus Real-Time PCR System (Life Technologies). The specific primers (Applied Biosystems, Foster City, CA, USA) used in this study are listed in **Supplementary Table S2** in the Supporting Information.

### Western blot analyses

The RIPA Lysis Buffer System (Santa Cruz Biotechnology Inc., Dallas, TX, USA) was used to lyse cells. Protein lysates (30 μg) were separated with SuperSep 6% gels (FUJIFILM Wako Chemical Corporation, Osaka, Japan) and incubated with Rabbit Anti-TRPA1 antibody (1:2000 dilution; ABN1009, Merck Millipore, Darmstadt, Germany) and β-Actin Mouse mAb (1:5000 dilution; 8H10D10; Cell Signaling Technology) as primary antibodies at 4 °C overnight. The membrane was washed and then incubated with secondary HRP-conjugated antibodies (GE Healthcare, Chicago, IL, USA). Immunoblotting was performed in accordance with the manufacturer’s instructions. Images were analyzed with ImageJ software. These assays were carried out as previously described (24).

### Air-liquid interface culture of primary bronchial epithelial cells

Primary bronchial epithelial cells were collected and cultured in accordance with previously described protocols (25, 26). Briefly, cells were obtained during routine fiberoptic bronchoscopy from never-smoker patients with normal lung function and pulmonary nodules by performing bronchial brushings from lobes without a pulmonary nodule. All bronchial brushings were obtained from the same anatomical region (bronchial generations 4-7). This study was conducted in accordance with the Declaration of Helsinki. The study protocol was approved by the Kyushu University Institutional Review Board for Clinical Research (2023-44), and all subjects provided written informed consent.

Cells were cultured in flasks coated with collagen (Cell Applications, Inc., San Diego, CA, USA) containing supplemented BEGM at 37°C in 5% CO_2_ and used for the air-liquid interface (ALI) culture within four passages. For ALI culture, cells were seeded onto collagen-coated Transwell inserts (0.33 cm^2^ Polyethylene terephthalate, 0.4 μm pore size; Corning, Glendale, AZ, USA) at a density of 1.0 × 10^5^ cells/cm^2^ with 200 μl apical volume and 500 μl basal volume of BEGM. After 48–72 h, the apical medium was removed, and the cells were maintained with 500 μl of PneumaCult™-ALI Maintenance Medium (STEMCELL Technologies, Tokyo, Japan) in the basal chamber. The medium was renewed every other day, and the monolayers were allowed to differentiate under the ALI condition for 21 days. Then, cells were treated with 1 µM papain alone, 100 µM AlTC alone, or a combination of both.

### Immunofluorescence staining

Differentiated cells were washed twice with PBS and fixed with 4% paraformaldehyde for 30 min. After blocking with PBS containing 1% bovine serum albumin (BSA) and 0.1% triton X-100 for 30 min at room temperature, the cells were incubated with rabbit anti-TSLP polyclonal antibody (20 μg/ml; Abcam, Cambridge, UK) prepared in PBS containing 1% BSA and 0.1% triton X-100 at 4°C overnight, followed by incubation with Alexa Fluor 488-conjugated goat anti-rabbit IgG antibody (diluted 1:500; Abcam) for 2 h at room temperature. Then F-actin and nuclear DNA were stained with Phalloidin-iFluor 647 Reagent (diluted 1:1000; Abcam) and 4’, 6-diamidino-2-phenylindole (DAPI) (diluted 1:1000; FUJIFILM Wako Chemical Corporation), respectively.

Fluorescent images were obtained using a Zeiss LSM 700 confocal microscope (Carl Zeiss, Jena, Germany) with a 63x oil 1.4 numerical aperture objective lens. Z-stack images were captured by taking an average of 15 slices at 1 μm intervals, ranging from the top, where F-actin can be observed, to the bottom. The images were processed using ZEN3.5 software (blue edition; Carl Zeiss), followed by further processing of the image using the section mode of Imaris Version 10.1 software (Bitplane AG, Zurich, Switzerland).

### Measurement of the intracellular calcium concentration

NHBE cells were incubated in BEGM at 37°C with 5% CO_2_ overnight to reach 80% confluence. Cells were then treated with 30 µM AITC. Intracellular calcium (Ca^2+^) concentrations were measured using the Calcium Kit-Fluo 4 (Dojindo, Kumamoto, Japan) following the manufacturer’s protocol. Briefly, cells were loaded with Fluo-4 dye and incubated at 37°C. Fluorescence was measured using the Infinite M200 fluorescence microplate reader (Tecan, Männedorf, Switzerland) at an excitation wavelength of 485 nm and an emission wavelength of 535 nm.

### Statistical analysis

Data are expressed as the mean ± SEM. Statistical differences among groups were analyzed using unpaired *t*-tests or an analysis of variance together with Tukey’s analysis. Data were analyzed with GraphPad Prism 8 software (GraphPad Software, La Jolla, CA, USA). *P* < 0.05 was considered significant.

## Results

### Effect of cold air exposure on papain-induced eosinophilic airway inflammation and the role of the TRPA1 channel

Papain was administered to both *Trpa1* KO mice and WT littermates (**Supplementary Figure S1A** in the Supporting Information). Papain treatment increased the numbers of total cells, eosinophils, and ILC2s in BALF (**Supplementary Figures S1B, C** in the Supporting Information); it also resulted in elevated IL-5 and IL-13 in BALF, along with the upregulated expression of these cytokines in the lungs of WT mice (**Supplementary Figure S1D**, E in the Supporting Information). There were no differences in the numbers of eosinophils and ILC2s and in these cytokine expressions between WT and *Trpa1* KO mice.

Cold air exposure alone did not alter the numbers of total cells, eosinophils, or ILC2s in BALF, nor did it affect the protein levels of IL-5 and IL-13 in BALF, in either *Trpa1* KO mice or WT littermates, compared with naïve mice. No significant differences were observed between WT and *Trpa1* KO mice (**Supplementary Figures S2A–E** in the Supporting Information).

To investigate whether exposure to cold air affects type-2 airway inflammation, papain-treated *Trpa1* KO mice and WT littermates were exposed to cold air (**Figure 1A**). In papain-treated WT mice, the cold air-exposed group exhibited significantly increased total cell, eosinophil, and ILC2 counts in BALF as compared to the room temperature group. Furthermore, histological examination revealed that papain exposure induced eosinophilic airway inflammation and mucus hypersecretion, which were further exacerbated by concomitant cold air exposure, showing enhanced epithelial response, inflammatory cell infiltration, and mucus production (**Figure 1B**). Conversely, in papain-treated *Trpa1* KO mice, no increases in the number of total cells, eosinophils, and ILC2s in BALF were observed under cold air exposure; these cell numbers were significantly lower than in papain-treated WT mice with cold air exposure (**Figures 1C, D**). In papain-treated *Trpa1* KO mice with cold air exposure, the protein expressions of IL-5 and IL-13 in BALF and the mRNA transcription of these cytokines in the lungs were also significantly suppressed as compared to WT mice (**Figures 1E, F**).

These results indicate that TRPA1 channels play an important role in the aggravation of papain-induced type-2 airway inflammation under cold air exposure.

### Activation of TRPA1 channels in airway epithelial cells is crucial for the release of TSLP

Next, we examined neuropeptides, CGRP and neuromedin U (NMU), which activate ILC2s as potential mediators of the mechanism by which cold air exposure aggravates papain-induced type-2 airway inflammation. These neuropeptides modulate ILC2 activation by binding to their respective receptors on ILC2s (27).

In papain-treated WT mice exposed to cold air, the expression of *Calca*, the gene encoding CGRP, was significantly upregulated compared to that in naïve mice, with a peak at 3 h post-treatment. At 24, 48, and 72 h, *Calca* expression decreased and was not significantly different from that in naïve mice (**Figure 2A**). Thus, both WT and *Trpa1* KO mice were treated with papain and exposed to cold air (**Figure 2B**). The expression of *Calca* was upregulated by papain stimulation but not by cold air exposure alone, and its expression was further enhanced by both stimuli were applied. This induction was observed regardless of the presence or absence of TRPA1 (**Figure 2C**, left).

On the other hand, papain administration tended to increase *Nmu* expression, although the difference did not reach statistical significance. However, additional cold air exposure did not further enhance *Nmu* expression in papain-treated mice (**Figure 2C** right).

We then examined the involvement of TRPA1 channels in the release of epithelial-derived cytokines. Papain administration significantly increased the amount of TSLP and IL-33 in BALF, as well as their mRNA expression in lung tissues in both WT and *Trpa1* KO mice. No significant differences were observed between WT and *Trpa1* KO mice. Cold air exposure alone did not affect the amount of TSLP or IL-33 in BALF or lung tissues in either WT or KO mice (**Supplementary Figure S3A**, **B** in the Supporting Information).

Both WT and *Trpa1* KO mice were treated with papain and exposed to cold air (**Figure 3A**). In papain-treated WT mice, the cold air-exposed group showed significantly increased amounts of TSLP in BALF and lung tissues compared to the room temperature group. In papain-treated *Trpa1* KO mice, no increase of TSLP expression was observed following cold air exposure (**Figures 3B, C**). As for IL-33, its levels were not elevated by cold air exposure, and no differences were observed between WT and *Trpa1* KO mice (**Figures 3B, C**).

These results suggest that the activation of TRPA1 channels by cold air exposure induces the release of TSLP from airway epithelial cells under papain stimulation, which may activate ILC2s and is associated with eosinophilic airway inflammation. Our findings highlight that TRPA1 functions not only in sensory neurons but also in airway epithelial cells, underscoring the novelty of epithelial TRPA1 as a contributor to cold-induced airway inflammation.

### The effect of a TRPA1 channel antagonist on papain-induced eosinophilic airway inflammation with cold air exposure

We investigated whether the pharmacological inhibition of TRPA1 channels could replicate the results observed in *Trpa1* KO mice. Papain-treated WT mice were administered HC030031, followed by cold air exposure (**Figure 4A**). The administration of HC030031 prevented an increase in the number of eosinophils and ILC2s observed in the untreated control group caused by the combination of papain treatment and cold air exposure (**Figures 4B, C**). It also prevented an increase in the concentration of IL-5 and IL-13 in BALF and the mRNA expression in lung tissues observed in the untreated control group (**Figures 4D, E**). Furthermore, HC030031 markedly suppressed the cold air-induced increase in TSLP expression, whereas it had no significant effect on IL-33 levels (**Figures 4F, G**).

These findings indicate that the pharmacological inhibition of TRPA1 channels replicates the results observed in *Trpa1* KO mice, indicating that TRPA1 channels are critically involved in the exacerbation of eosinophilic airway inflammation mediated by innate immunity with cold air exposure.

### TRPA1-mediated Ca^2+^ signaling drives TSLP production in airway epithelial cells

**Figure 5:**
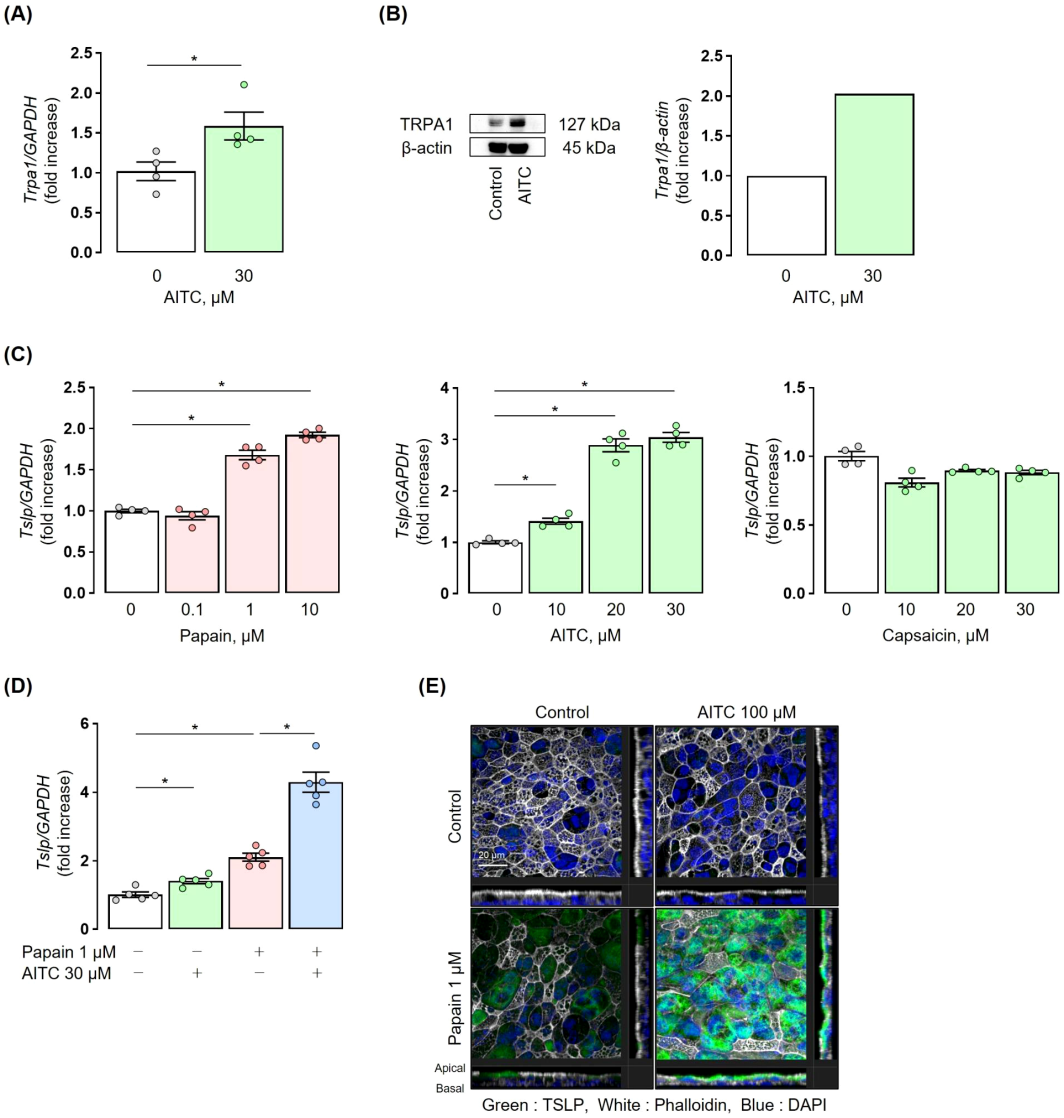
TRPA1 channels induce TSLP expression in human airway epithelial cells. **(A)** NHBE cells were incubated with 30 µM AITC for 3 h, resulting in upregulation of TRPA1 mRNA. **(B)** NHBE cells were incubated with 30 µM AITC for 6 h. Western blot images were analysed using ImageJ software. Western blot analysis showed the presence of TRPA1 in NHBE cells and its increased expression. **(C)** NHBE cells were incubated with 0.1-10 µM papain for 6 h or 10-30 µM AITC for 3 h. Treatment with papain and AITC upregulated TSLP mRNA expression in NHBE cells in a concentration-dependent manner, respectively. Treatment with 10-30 µM capsaicin for 3 h did not significantly increase TSLP mRNA expression. **(D)** Co-stimulation with 30 µM AITC and 1 µM papain for 6 h upregulated TSLP mRNA expression in NHBE cells, as compared to papain or AITC alone. **(E)** Immunofluorescence staining using ALI culture showed that co-stimulation with 100 µM AITC and 1 µM papain for 12 h induced a marked enhancement in green fluorescence, as compared to papain or AITC alone, indicating increased TSLP production. Fluorescent images were obtained using a Zeiss LSM 700 confocal microscope with a 63x oil immersion objective (NA 1.4). The final displayed magnification is 250x. Scale bar: 20 µm. TRPA1 and TSLP expression were measured by real-time PCR **(A, C, D)**. Mean ± SEM: n = 4–6 wells per group; data shown are from a representative experiment out of three independent experiments with similar results. Unpaired *t-*tests with Tukey’s *post hoc* test; *P < 0.05. ALI, Air-liquid interface.

To elucidate how TRPA1 channel activation leads to TSLP expression in airway epithelial cells, we conducted *in vitro* experiments using normal human bronchial epithelial (NHBE) cells. TRPA1 mRNA was detected in NHBE cells, and its expression was upregulated following treatment with allyl isothiocyanate (AITC), a TRPA1 channel agonist (**Figure 5A**). Western blot analysis also confirmed the presence of TRPA1 protein in NHBE cells and showed increased expression following AITC treatment (**Figure 5B**). Stimulation with either papain or AITC upregulated TSLP expression in NHBE cells in a concentration-dependent manner. Since AITC can activate both TRPA1 and TRPV1 channels, we investigated whether the AITC-induced upregulation of TSLP is mediated specifically through TRPA1 activation. To this end, we treated NHBE cells with capsaicin, a selective TRPV1 agonist, at the same concentration used for AITC. Capsaicin treatment did not significantly increase TSLP expression, suggesting that TRPV1 is not involved in this response and supporting the role of TRPA1 in mediating TSLP induction (**Figure 5C**).

Co-stimulation with papain and AITC synergistically increased the expression of TSLP in NHBE cells as compared to stimulation with either papain or AITC alone (**Figure 5D**). Immunofluorescence staining in ALI cultures of human bronchial epithelial cells showed a significant increase in green fluorescence with the co-stimulation of papain and AITC as compared to stimulation with either papain or AITC alone, confirming the synergistic induction of TSLP under co-stimulation (**Figure 5E**).

**Figure 6:**
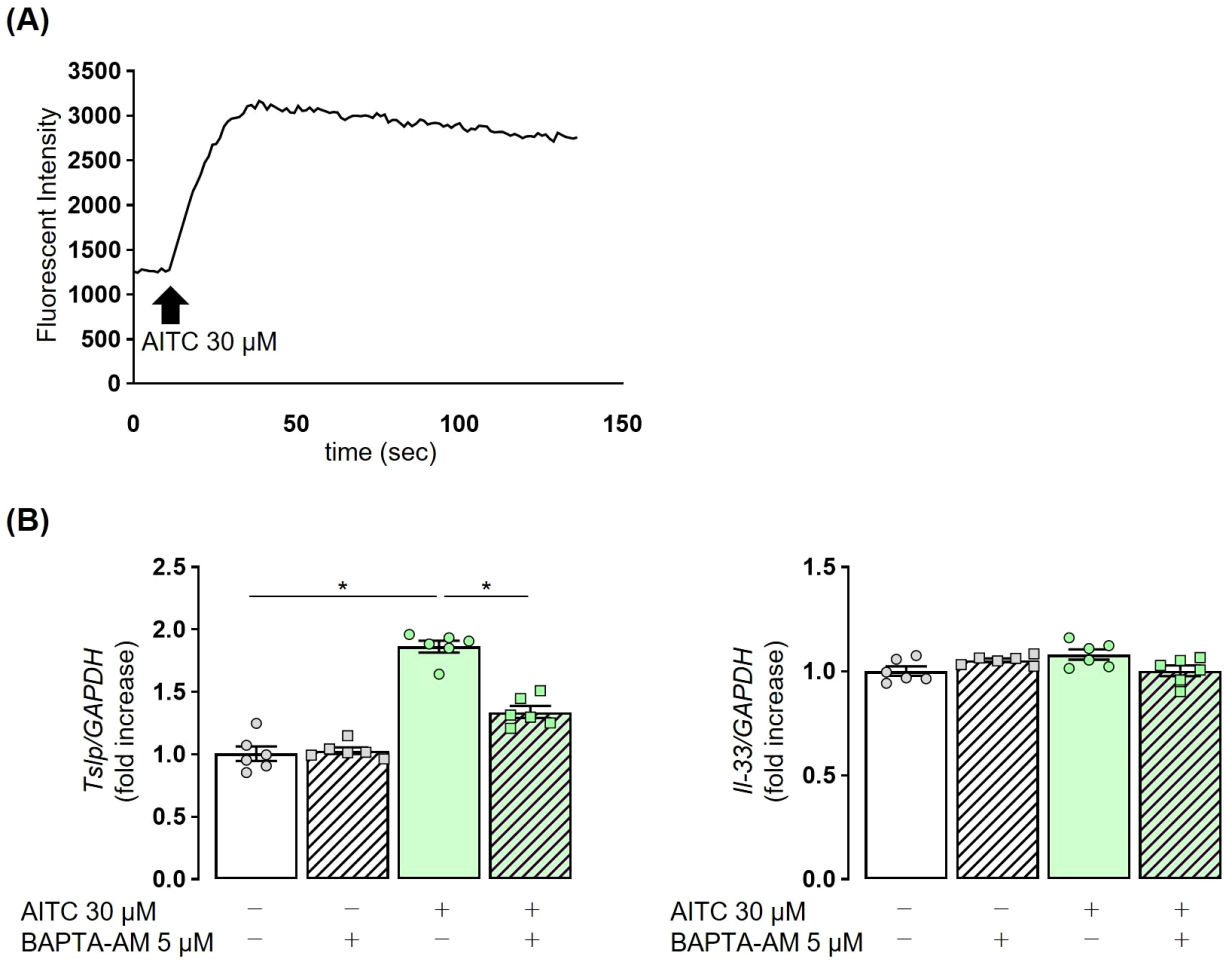
TRPA1-induced TSLP release in NHBE cells is dependent on intracellular Ca^2+^. **(A)** The administration of 30 µM AITC in NHBE cells resulted in an increase in fluorescence intensity, indicating elevated intracellular calcium levels. **(B)** The elevated expression of TSLP in NHBE cells treated with 30 µM AITC was reduced following the addition of 5 µM BAPTA-AM, a calcium chelator, for 3 h Fluorescence intensity was measured by the Calcium Kit-Fluo-4 **(A)**. TSLP and IL-33 expression were measured by real-time PCR **(B)**. Mean ± SEM: n = 4–6 wells per group; data shown are from a representative experiment out of three independent experiments with similar results. One-way ANOVA with Tukey’s *post hoc* test; *P < 0.05.

Finally, we examined the mechanism by which TRPA1 channel stimulation enhances TSLP expression in airway epithelial cells by focusing on changes in intracellular Ca^2+^. AITC was added to the culture medium of NHBE cells, and intracellular Ca^2+^ concentrations were measured. An obvious increase in fluorescence intensity was observed, indicating elevated intracellular Ca^2+^ concentrations (**Figure 6A**). AITC-stimulated NHBE cells were treated with the calcium chelator BAPTA-AM. A significant reduction in TSLP expression was observed in the BAPTA-AM-treated group as compared to controls. In contrast, AITC stimulation had no effect on IL-33 expression, and this remained unchanged with calcium chelation (**Figure 6B**). These findings demonstrate that in airway epithelial cells, the Ca^2+^-mediated signaling pathway is essentially involved in the TRPA1-mediated TSLP release.

## Discussion

In this study, we revealed the important role of TRPA1 channels in papain-induced murine asthma models. First, the activation of TRPA1 under cold air exposure results in increased eosinophils and ILC2s as well as type-2 cytokines in BALF. Second, in this model, such type-2 inflammation is induced by TRPA1-mediated TSLP expression. Third, papain stimulation and TRPA1 channel activation work together to increase the expression of TSLP. Finally, the increase in TSLP expression via the TRPA1 channel is intracellular Ca^2+^ dependent.

Although asthma exacerbation induced by cold air exposure has a certain prevalence worldwide, its molecular pathogenesis remains unknown, and no specific treatment has been defined. This is due to the lack of molecular biological analyses in animal models that mimic this condition. In this study, we developed a novel asthma model by combining papain stimulation with intermittent cold air exposure at 4°C. Using this model, we have shown that the activation of TRPA1 channels is specifically involved in the exacerbation of innate immunity-mediated eosinophilic airway inflammation under cold air exposure; these findings provide a molecular mechanistic explanation for such asthma exacerbation.

TRP channels act as cellular sensors that respond to various environmental changes, including changes in temperature, pressure, chemicals, oxidative stress, osmolarity, and pH (28). They are expressed in nearly all cell types and are vital to numerous homeostatic processes (29). Initially, the TRPA1 channel, a member of the TRP subfamily, was thought to be predominantly expressed in sensory neurons. However, numerous studies have revealed that TRPA1 channels are widely expressed in non-neuronal cells, such as epithelial cells, fibroblasts, and mast cells (30). Previous researches have confirmed the presence of TRPA1 channels in the airway epithelium (31–34). In the airway, TRPA1 channels are crucial for detecting irritant chemicals and hypoxia, triggering escape behaviors, and modulating respiratory responses (35).

Our results demonstrated that cold air exposure significantly exacerbated eosinophilic airway inflammation in a papain-treated murine model, and this inflammation was ameliorated in *Trpa1* KO mice or WT mice treated with a TRPA1 channel antagonist (**Figures 1**, **4**). Previous studies have linked TRPA1 channels to the development of eosinophilic airway inflammation in OVA-induced murine models (16), and cold air stimulation may further aggravate such inflammation by activating TRPA1 channels (33, 36). Generally, OVA-induced eosinophilic airway inflammation is the result of acquired immunity, driven primarily by antigen-specific Th2 cells. In contrast, the papain-induced eosinophilic airway inflammation model we used highlights the crucial role of innate immunity, particularly ILC2 cells (19). Therefore, the exacerbation of airway inflammation resulting from exposure to cold temperatures is triggered by TRPA1 channels that sense changes in the environmental temperature, via both innate and acquired immune responses. However, the molecular mechanisms of how innate and acquired immunity function distinctively in the process of airway inflammation through TRPA1 channels were not examined in this study and require further investigation.Previous research has linked TRPA1 to the release of neuropeptides (16). The sensory nerves in the airway express receptors for alarmins and cytokines. Upon stimulation, they release neuropeptides, such as CGRP, that mediate vasodilation, bronchoconstriction, and mucus production (37, 38). ILC2s also express receptors for both NMU and CGRP, and these neuropeptides are known to promote ILC2-mediated eosinophilic airway inflammation (27, 39, 40). Thus, we focused on neuro-immune crosstalk in our model (37, 41), and we observed a significant upregulation of *Calca* in models exposed to both papain and cold air, regardless of the presence of TRPA1 (**Figure 2**). Previous reports have shown that OVA-induced allergic inflammation combined with ozone exposure exacerbated airway inflammation via the TRPV1 channel, another member of the TRP channel family, which was associated with elevated CGRP levels (42). In contrast, under papain and cold air exposure, the mechanism of increased CGRP expression appeared to be independent of TRPA1, and we did not functionally examine its role in ILC2 activation. Therefore, the contribution of CGRP to the exacerbation of type-2 airway inflammation remains uncertain and should be clarified in future studies.

TSLP and IL-33 are released from airway epithelial cells and are involved in the induction of type-2 innate immunity (43). In a papain-treated murine model, we previously reported elevated protein expressions of IL-33 and TSLP in BALF at 3 h post-administration, which subsided after 24 h (21). In the present study, only TSLP expression increased further in models exposed to both papain and cold air (**Figure 3**). These results suggest that cold air-induced activation of TRPA1 channels may promote the additional secretion of TSLP from airway epithelial cells, leading to ILC2 activation and the worsening of eosinophilic airway inflammation. It should also be noted that following a single papain plus cold exposure, we observed early increases in upstream mediators, such as epithelial and neuropeptide transcripts, but no downstream increases in IL-5/IL-13 or BALF eosinophils or ILC2 numbers were detected. This finding suggests that repeated exposures are required for the full development of type-2 airway inflammation. This supports the concept that initial epithelial activation is an early step preceding overt inflammatory cell recruitment.

We observed that the co-stimulation of human airway epithelial cells with the TRPA1 agonist AITC, not TRPV1 agonist capsaicin with papain resulted in a synergistic increase in TSLP expression at 37°C (**Figure 5**). In addition, we found that the increased intracellular Ca^2+^ concentration through the activation of TRPA1 channels is implicated in the production of TSLP in airway epithelial cells (**Figure 6**), which are similar to previous reports for TRPV1 (44, 45). Considering the fact that we observed lower Ct values for TRPV1 than for TRPA1 in NHBE cells (data not shown), possible explanation for our results is that the activation signaling of TRP family is determined by cell types as well as activation conditions. It has been well known that *in vivo*, TRPA1 is activated by low temperatures, whereas TRPV1 requires stimulation at high temperatures above 43°C to be activated (46). Therefore, we conclude in this study the TRPA1 is the primary mediator of TSLP induction in response to cold exposure, potentially contributing to asthma exacerbations. Additionally, activation of the TRPA1 channel has been reported to promote TSLP release through a Ca^2+^/NFAT pathway in nasal epithelial cells (45). Although the precise mechanisms how NFAT regulates TSLP transcription remain to be elucidated, our findings raise the possibility that a similar Ca^2+^/NFAT-dependent pathway operates downstream of TRPA1 in airway epithelial cells, which warrants further investigation. Regarding IL-33, its expression was not affected by TRPA1 activation in our study. We speculate for this differential regulation that IL-33 is constitutively expressed in airway epithelial cells, and TRPA1 activation may have a limited effect on its expression (47).

Together, our *in vivo* mouse model and *in vitro* human epithelial cell experiments provide complementary evidence for the proposed cold-TRPA1-TSLP axis. In mice, cold exposure exacerbated papain-induced eosinophilic inflammation in a TRPA1-dependent manner, whereas in human NHBE cells, TRPA1 activation directly enhanced TSLP production. These findings bridge the two systems and support the translational relevance of TRPA1-mediated epithelial cytokine release as a key mechanism underlying cold air-induced asthma exacerbation.

In this study, we used both commercially available NHBE cells and primary ALI cultures. While NHBE cells allowed reproducible pharmacological analyses, TSLP protein levels in culture supernatants were below the detection limit. To complement this, we employed ALI cultures, which better mimic differentiated airway epithelium, to confirm TSLP production by immunofluorescence. In ALI cultures, TSLP protein was scarcely detected after AITC stimulation and did not differ from the control. This finding is consistent with our *in vivo* results, in which cold exposure alone (TRPA1 stimulation) did not increase TSLP, whereas papain induced a significant increase that was further enhanced by cold stimulation. We interpret these results to reflect differences in epithelial differentiation and the requirement of an inflammatory context for robust TSLP protein production. Submerged NHBE cells mainly represent basal-like cells, in which TSLP mRNA is readily induced by TRPA1 activation, but protein synthesis is minimal, consistent with previous reports using 2D airway epithelial models. In contrast, ALI cultures recapitulate a differentiated, polarized airway epithelium. Our Z-stack immunofluorescence analyses demonstrated that TSLP protein localizes predominantly to the apical side of ciliated epithelial cells, suggesting that although basal and parabasal cells show higher transcript levels in single-cell RNA-seq datasets (48), actual protein synthesis and secretion occur mainly in differentiated ciliated cells. Thus, the apparent discrepancy between NHBE and ALI cultures can be explained by cell-type specific expression patterns and differentiation status, and importantly, the ALI findings reinforce our *in vivo* observations that TRPA1 stimulation alone is insufficient for TSLP protein induction without additional inflammatory stimuli.

The administration of the TRPA1 antagonist HC030031 effectively mitigated the cold air-induced exacerbation of ILC2-mediated eosinophilic airway inflammation (**Figure 4**). Several TRP channels, including TRPA1, are being explored as pharmacological targets for asthma (13, 29, 49); however, what type of asthma TRPA1 is specifically involved in has remained unclear, and no drug targeting TRPA1 channels has been clinically developed. Our results provide scientific evidence to clarify the potential of TRPA1 antagonists as a therapeutic agent for cold air-induced asthma exacerbation.

In this study, we were unable to detect consistent changes in TRPA1 expression in whole lung tissues after cold and/or papain exposure, likely because TRPA1 is confined to limited cell subsets; prior reports (31–34) and LungMAP transcriptomic data (50) support its presence in airway epithelium but largely within specific populations such as pulmonary neuroendocrine cells. Our immunostaining attempts also failed to yield reproducible epithelial signals. While several studies have reported TRPA1 expression in airway epithelium (31) or upregulation following OVA-induced allergic inflammation (33), the staining patterns appear highly variable. Considering the biological differences between adaptive (OVA) models previously reported and our innate (papain) model, these findings support the notion that, in our system, activation of TRPA1 rather than a significant increase in its expression is the key trigger of airway inflammation. We did not observe papain-induced increases in TRPA1 expression in NHBE cells. We also made preliminary attempts to expose NHBE cells to cold conditions, but due to technical limitations in our facility, we could not obtain consistent results regarding TRPA1 expression. Therefore, we cannot completely exclude the possibility that cold stress upregulates TRPA1 in human airway epithelium. Taken together, a plausible explanation for the synergistic increase in TSLP with papain plus cold stimulation is that papain elicits epithelial stress responses (e.g., protease-activated receptor signaling) that prime the transcriptional machinery, while TRPA1 activation provides a convergent Ca^2+^ influx that enhances NFAT activity and thereby augments TSLP transcription. This model explains why cold alone has little effect, yet markedly boosts papain-evoked TSLP, and it highlights specific, testable mediators for future investigation.

One limitation of this study is that the *in vitro* experiments were conducted using airway epithelial cells from healthy subjects. Second, it was not feasible to perform *in vitro* experiments with cells exposed to a cold environment as already discussed. Therefore, we used the TRPA1 agonist AITC as an alternative approach in **Figures 5** and **6** to focus on demonstrating whether activation of TRPA1 leads to TSLP production, an unprecedented cytokine involved in cold stress-induced airway inflammation, in human airway epithelial cells. This approach confirmed the functional presence of the TRPA1-TSLP axis in human cells; however, *in vivo* and *in vitro* responses differed. Specifically, cold exposure alone did not induce TSLP expression *in vivo* but did so in the presence of papain, whereas AITC alone was sufficient to induce TSLP in NHBE cells. This discrepancy may reflect differences in the mode of TRPA1 activation: AITC is a potent and direct agonist, while cold stress alone appears insufficient to induce TRPA1 activation, and may instead act indirectly through endogenous agonists such as reactive oxygen species or lipid peroxidation products (51, 52). Although we did not quantify these mediators in the present study, we consider this an important future direction to further substantiate the cold-TRPA1 axis. This issue is further complicated by species-specific differences in TRPA1 sensitivity to cold stimulation: TRPA1 was initially identified as a cold sensor in mice (17), whereas subsequent studies have indicated that human TRPA1 is less sensitive to such condition (53). Nevertheless, under certain conditions such as hypoxia, cold-induced production of reactive oxygen species can activate human TRPA1, suggesting that our observations are still relevant to cold-induced airway inflammation in humans (51). Finally, all mice were maintained at 22 °C, which is below the thermoneutral zone (28-32 °C) and may impose chronic cold stress affecting immune and inflammatory responses (54, 55). The absence of a thermoneutral (30 °C) control group is therefore a limitation of this study, and environmental factors such as cage density or elevated ambient temperature may also modulate airway responses.

In conclusion, our data demonstrate that TRPA1 channels contribute to ILC2-dependent airway inflammation under cold air exposure by promoting TSLP production through an increase in intracellular calcium influx in airway epithelial cells. These findings offer valuable insights into the novel role of TRPA1 channels in modulating the production of epithelial-derived cytokines, such as TSLP, in response to environmental temperature changes and suggest specific therapeutic targets for eosinophilic airway inflammatory disorders.

## Data Availability

The raw data supporting the conclusions of this article will be made available by the authors, without undue reservation.
