# Supplementary material for: Cold temperature enhances innate eosinophilic airway inflammation via transient receptor potential ankyrin1
Source: Front Immunol. 2025 Nov 13;16:1655919. doi: 10.3389/fimmu.2025.1655919 (PMC12657488; doi:10.3389/fimmu.2025.1655919)
Supplement: Supplementary file 1 [file DataSheet1.docx]

Supplementary Material

**Supplemental Figure S1. Papain induces airway eosinophilic inflammation, with no discernible distinction observed between WT and *Trpa1* KO mice.**

A, Experiment protocol: Airway inflammation was induced by the administration of papain for 3 consecutive days. B, C, D, E, The numbers of total cells, eosinophils (B), and ILC2s (C), and the production of IL-5 and IL-13 in BALF (D) and the expression of Il-5 and Il-13 in lung tissues (E) were increased by the administration of papain. There were no differences in these findings between WT and *Trpa1* KO mice. The number of ILC2s in BALF was measured using flow cytometry (C). IL-5 and IL-13 production and expression were measured by ELISA (D) and real-time PCR (E). Mean ± SEM: n = 4–6 for each group; two-way ANOVA with Tukey's post hoc test; **P* < 0.05. Data are representatives of three independent experiments. i.n., intranasal


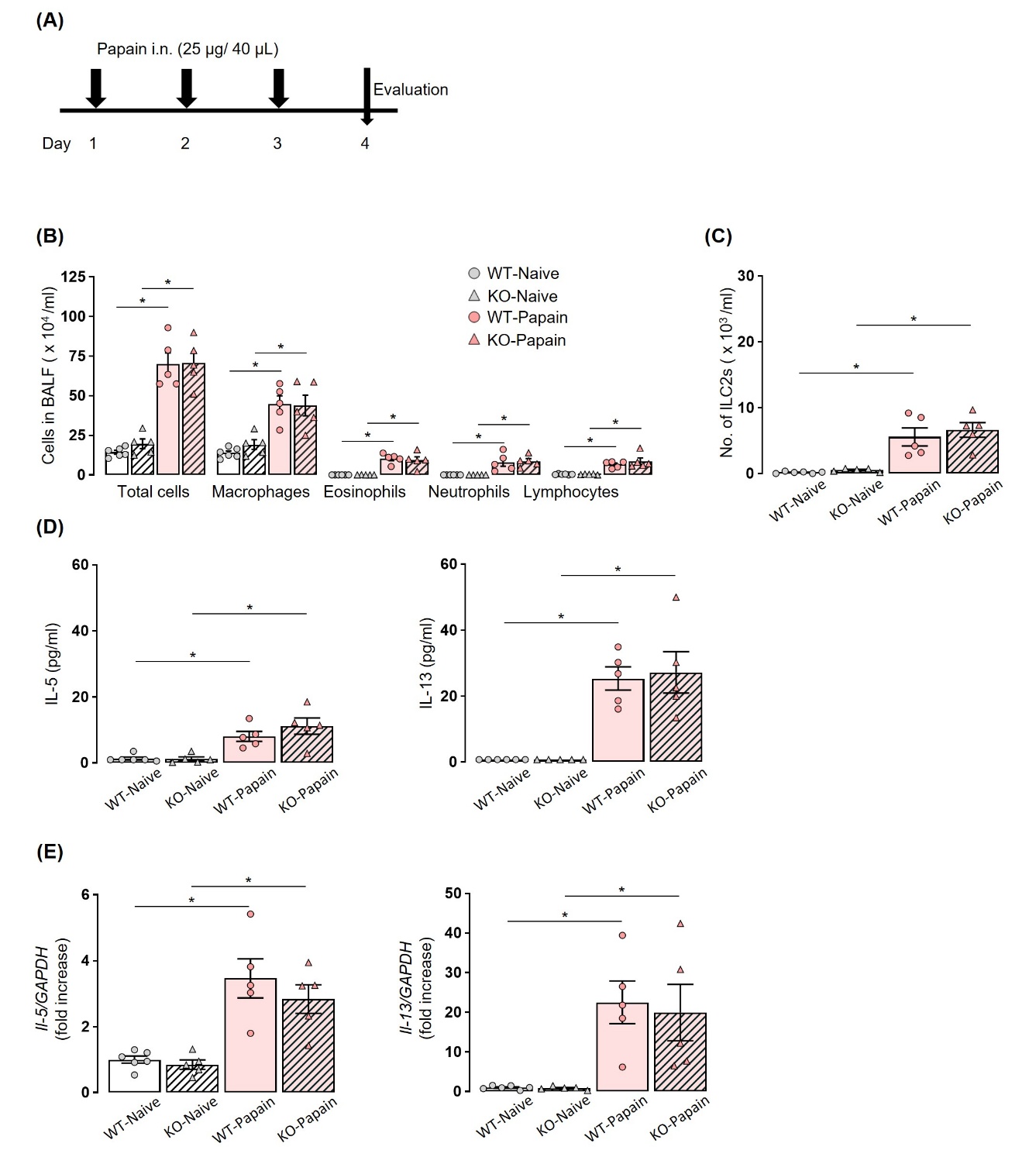


**Supplemental Figure S2. Cold air exposure alone does not induce airway eosinophilic inflammation.**

A, Experiment protocol: Mice were kept at 4°C for 8 h, followed by at 22°C for 16 h for 3 consecutive days. B, C, D, E, The numbers of total cells, eosinophils (B), and ILC2s (C), and the production of IL-5 and IL-13 (D) in BALF and the expression of Il-5 and Il-13 in lung tissues (E) were not increased by cold air exposure alone. There were no differences in these findings between WT and *Trpa1* KO mice. The number of ILC2s in BALF were measured using flow cytometry (C). IL-5 and IL-13 production and expression were measured by ELISA (D) and real-time PCR (E). Mean ± SEM: n = 4–6 for each group; two-way ANOVA with Tukey's post hoc test; **P* < 0.05. Data are representatives of two independent experiments. i.n., intranasal


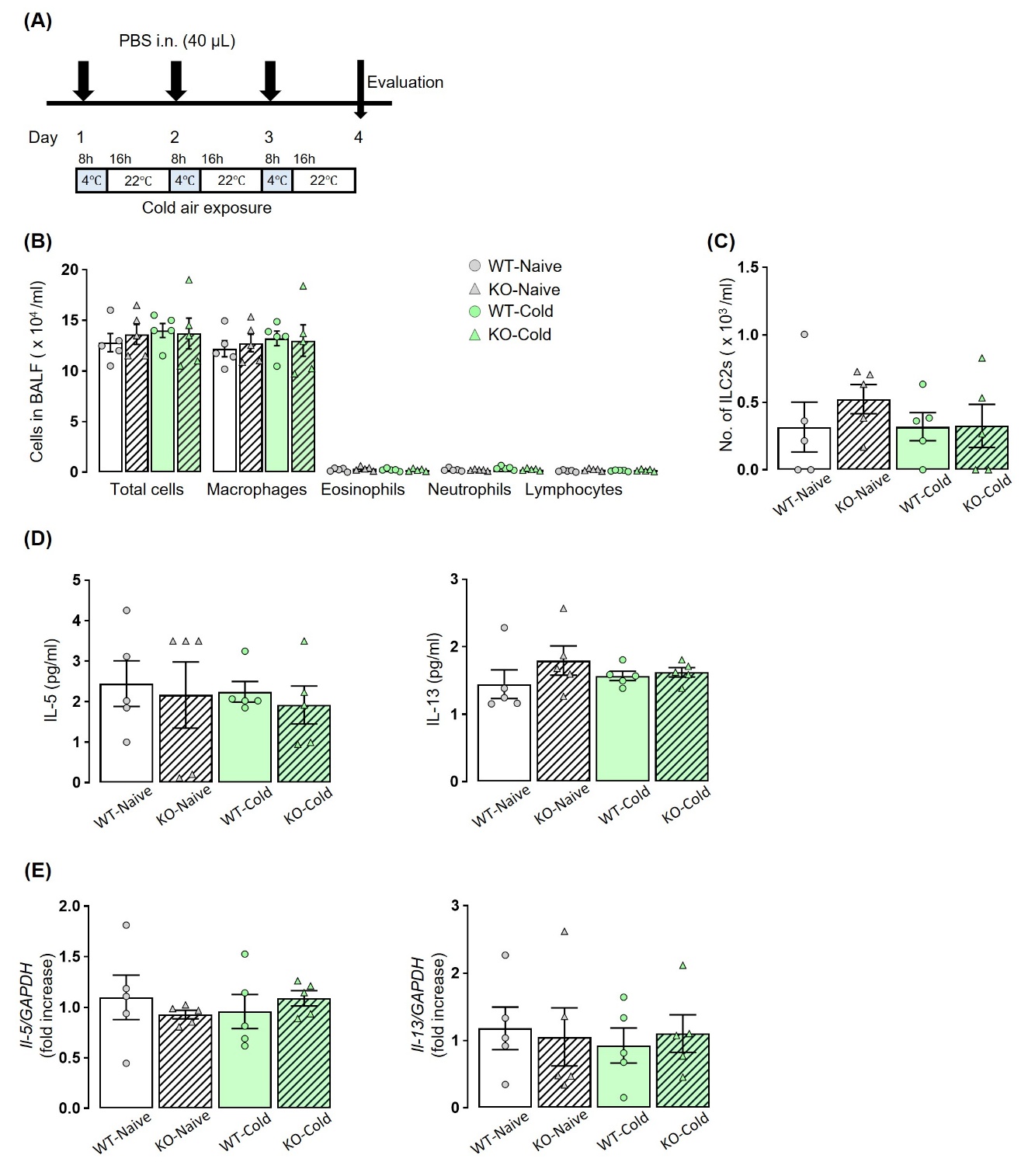


**Supplemental Figure S3. Papain increases the levels of epithelium-derived cytokines, whereas cold exposure does not.**

A, B, Papain administration increased the production of epithelium-derived cytokines in BALF (A) and their expression in lung tissues (B). These findings were not increased by cold air exposure alone. There were no differences in these findings between WT and *Trpa1* KO mice. TSLP and IL-33 production and expression were measured by ELISA (A) and real-time PCR (B). Mean ± SEM: n = 4–6 for each group; two-way ANOVA with Tukey's post hoc test; **P* < 0.05. Data are representatives of two independent experiments.


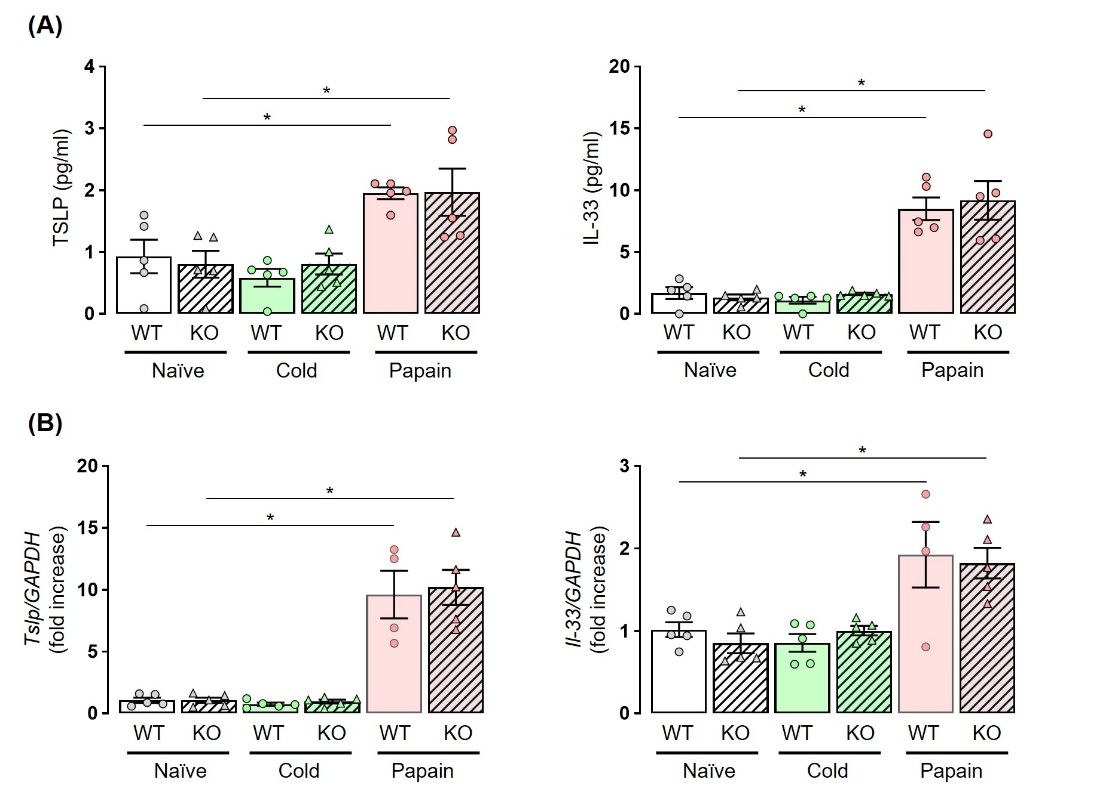


**Supplemental Table S1. A list of antibody sources**

| Antigen | Clone | Label | Source |
| --- | --- | --- | --- |
| Lineage cocktail  (CD3ε, CD11b, CD45RA/B220,  Ly-76, Ly-6G/Ly-6C) | 145-2C11, M1/70, RA3-6B2, TER-119, RB6-8C5 | PerCP-Cy5.5  FITC | BD bioscience  Biolegend |
| T1/ST2 | U29-93 | BV421  PE | BD bioscience |
| KLRG1 | 2F1 | BV650 | BD bioscience |
| Thy1.2 | 30-H12 | BV786  FITC | BD bioscience  BioLegend |
| CD45.2 | 104 | PE-Cy7 | BD bioscience |

**Supplemental Table S2. A list of primers used in quantitative real-time PCR**

| Primer | Assay ID; Applied Biosystems |
| --- | --- |
| Mouse *Gapdh* | Mm99999915_g1 |
| Mouse *Il-5* | Mm00439646_m1 |
| Mouse *Il-13* | Mm00434204_m1 |
| Mouse *Calca* | Mm00801463_g1 |
| Mouse *Nmu* | Mm00479868_m1 |
| Mouse *Il-33* | Mm00505403_m1 |
| Mouse *Tslp* | Mm01157588_m1 |
| Human *Gapdh* | Hs99999905_m1 |
| Human *Trpa1* | Hs00175798_m1 |
| Human *Tslp* | Hs00263639_m1 |
